# Evaluation of Pattern Recognition and Feature Extraction Methods in ADHD Prediction

**DOI:** 10.3389/fnsys.2012.00068

**Published:** 2012-09-24

**Authors:** João Ricardo Sato, Marcelo Queiroz Hoexter, André Fujita, Luis Augusto Rohde

**Affiliations:** ^1^Center of Mathematics, Computation and Cognition, Universidade Federal do ABCSanto Andre, Brazil; ^2^Laboratório Interdisciplinar de Neurociências Clínicas, Department of Psychiatry, Universidade Federal de São PauloSão Paulo, Brazil; ^3^Department of Computer Science, University of São PauloSão Paulo, Brazil; ^4^Attention Deficit/Hyperactivity Disorder Outpatient Program, Child and Adolescent Psychiatric Division, Hospital de Clínicas de Porto AlegrePorto Alegre, Brazil; ^5^Instituto Nacional de Psiquiatria do DesenvolvimentoSão Paulo, Brazil

**Keywords:** ADHD, machine learning, SVM, classification, diagnosis, prediction, features

## Abstract

Attention Deficit/Hyperactivity Disorder (ADHD) is a neurodevelopmental disorder, being one of the most prevalent psychiatric disorders in childhood. The neural substrates associated with this condition, both from structural and functional perspectives, are not yet well established. Recent studies have highlighted the relevance of neuroimaging not only to provide a more solid understanding about the disorder but also for possible clinical support. The ADHD-200 Consortium organized the ADHD-200 global competition making publicly available, hundreds of structural magnetic resonance imaging (MRI) and functional MRI (fMRI) datasets of both ADHD patients and typically developing (TD) controls for research use. In the current study, we evaluate the predictive power of a set of three different feature extraction methods and 10 different pattern recognition methods. The features tested were regional homogeneity (ReHo), amplitude of low frequency fluctuations (ALFF), and independent components analysis maps (resting state networks; RSN). Our findings suggest that the combination ALFF+ReHo maps contain relevant information to discriminate ADHD patients from TD controls, but with limited accuracy. All classifiers provided almost the same performance in this case. In addition, the combination ALFF+ReHo+RSN was relevant in combined vs. inattentive ADHD classification, achieving a score accuracy of 67%. In this latter case, the performances of the classifiers were not equivalent and L2-regularized logistic regression (both in primal and dual space) provided the most accurate predictions. The analysis of brain regions containing most discriminative information suggested that in both classifications (ADHD vs. TD controls and combined vs. inattentive), the relevant information is not confined only to a small set of regions but it is spatially distributed across the whole brain.

## Introduction

Attention Deficit/Hyperactivity Disorder (ADHD) is a worldwide prevalent disorder (Polanczyk et al., 2007) and is characterized by excessive childhood onset inattention, hyperactivity, and impulsivity (American Psychiatric Association, 1994) that usually persists into adulthood (Mannuzza and Klein, 2000).

In recent years, structural magnetic resonance imaging (MRI) and functional MRI (fMRI) techniques have been extensively used in the quantitative analysis of the brain in healthy individuals and patients with psychiatric disorders in an attempt to increase our understanding of human brain structural and functional networks (Bassett and Bullmore, 2009; Biswal et al., 2010). In comparison to typically developing (TD) individuals, structural neuroimaging studies have shown that ADHD patients present abnormalities in several regions including the frontal, parietal, and occipital lobes, the basal ganglia and the cerebellum (Castellanos et al., 1996; Sowell et al., 2003; Seidman et al., 2006). Abnormal brain activation in the dorsolateral prefrontal cortex, inferior prefrontal cortex, dorsal anterior cingulate cortex, basal ganglia, thalamus, and parietal cortex is also observed in ADHD compared to controls (Dickstein et al., 2006).

However, despite the enormous increase in the number of studies using structural and fMRI in the last two decades, applications in clinical practice and reliability in disease diagnosis are still not well established. Recently, pattern recognition approaches and classifiers based on machine learning were proposed for the extraction of predictive information of structural and fMRI data (Fan et al., 2005). Misra et al. (2009) explored the clinical value of structural MRI and pattern recognition methods focusing on Alzheimer diagnosis and prognosis in mild cognitive impaired patients. Ecker et al. (2010) have shown that Support Vector Machines (SVM) combined with computational neuroanatomy can be useful in autism diagnosis support, achieving a discrimination accuracy of 90%. Sato et al. (2011) investigated the potential of structural MRI as a clinical tool to identify individuals with psychopathy. These methods have the potential to translate objective biological information extracted from neuroimaging data to clinical practice (Marquand et al., 2008; Zhu et al., 2008; Dosenbach et al., 2010).

In 2011, the ADHD-200 Consortium, a self-organized, grassroots initiative, dedicated to accelerating the scientific community’s understanding of the neural basis of ADHD through the implementation of discovery-based science, launched the “ADHD-200 Global Competition”^1^ in which researchers around the world were invited to develop diagnostic classification tools for ADHD diagnosis based on structural MRI and fMRI of the brain. The ADHD-200 Consortium made available datasets aggregated across eight independent imaging sites that contain resting state fMRI and structural MRI datasets for individuals diagnosed with ADHD and TD individuals.

The interdisciplinary research combining classifiers based on machine learning and clinical applications of neuroimaging has been shown to be fruitful and innovative. Although several pattern recognition classifiers are available in the technical literature, there is not a single optimal classifier for the general case. Furthermore, there is no single learning algorithm which always lead to the most accurate learner in all domains. The most usual approach is to evaluate several classifiers and to choose the one with the best performance on a independent validation set (Alpaydin, 2010, chapter 17). Different classifiers may perform better than others depending on the neurological/neuropsychiatric disease or the data modality available. In addition, there are a number of different features (the predictor variables used for classification) that can be extracted from resting state fMRI data. Three of the most frequently used features are the regional homogeneity maps (ReHo, Zang et al., 2004), fractional amplitude of low frequency fluctuations (ALFF) maps (fALFF, Zou et al., 2008), and independent component maps (ICA, Smith et al., 2009).

In the current study, we present a systematic evaluation of the classification performance of 10 different pattern recognition classifiers combined with three feature extraction methods. The features explored in combination with these classifiers were the ReHo, fALFF, and ICA maps. We evaluated the classification accuracies for typical controls vs. ADHD patients and also for inattentive vs. combined ADHD groups. The aims of the current study are the following: (i) to estimate the accuracy measures of classification (controls vs. ADHD and inattentive vs. combined ADHD) based on a large resting state fMRI dataset; (ii) to compare the prediction accuracy of different classifiers and/or penalty functions; (iii) to identify brain regions containing discriminant information between groups. The novel findings of this study are that most classifiers have approximately the same performance and that the discriminant information is not concentrated in a small set of regions but distributed in the entire brain.

## Materials and Methods

**Figure 1:**
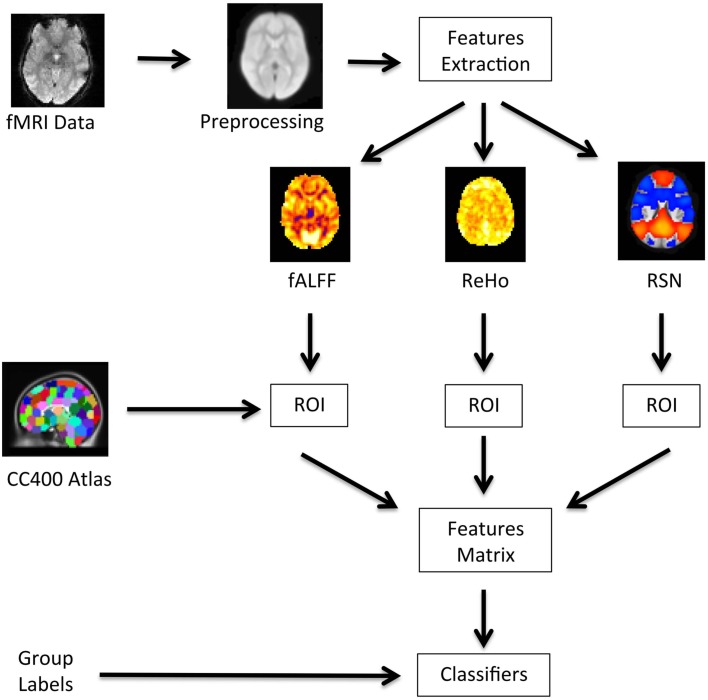
**Flow of data processing**. The raw fMRI data is preprocessed; the feature maps for fALFF, ReHo, and RSN are obtained; the average coefficient of these maps within each ROI are calculated using the CC400 atlas; the data is then organized in a features matrix which is then input to the classifiers.

A diagram showing a summary of the analysis pipeline is presented in Figure 1. Basically, after preprocessing the fMRI data, we extracted the fALFF, ReHo, and resting state networks (RSN) maps, and calculate the average values of these maps for each region. The regions were defined by a functional atlas and were used as an input to the classifiers.

### Participants and data

The data used in the current study was acquired by the ADHD-200 Consortium which provided public release of 929 resting state scans of children and adolescents with ADHD and typical controls. The data was acquired in eight different sites: Peking University, Bradley Hospital/Brown University, Kennedy Krieger Institute, NeuroIMAGE Sample, New York University Child Study Center, Oregon Health & Science University, University of Pittsburgh, and Washington University. This data was released in the ADHD-200 Competition, coordinated by Prof. Damien Fair and Prof. Michael Milham, and organized by the Consortium. The competition released both the structural MRI and fMRI scans of 759 subjects with the respective labels (TD control, inattentive, and combined ADHD) in order to motivate participants to train and develop algorithms to predict the group of a single subject, given his/her neuroimaging data. The data of additional 170 subjects was then released without the group labels and the competition participants were asked to send the predicted labels. The true label of this test set of subjects was then released after the announcement of the competition results. All research conducted by ADHD-200 contributing sites was conducted with local Internal Review Board approval, and contributed in compliance with local Internal Review Board protocols. All data distributed via the International Neuroimaging Data sharing Initiative is fully anonymized in compliance with the HIPAA Privacy Rules. Prior to release, it is ensured that the 18 patients’ identifiers were removed, as well as face information. The complete demographic data is presented in Table 1. Further details about both the samples and scanning parameters can be obtained under request to the ADHD-200 consortium.

**Table 1:** **Demographic information of the subjects from the ADHD-200 sample**.

Group	*N*	Males	Mean age (SD) – years
TD controls	546	286 (52.4%)	12.29 (3.46)
Combined	249	193 (77.5%)	11.21 (3.02)
Inattentive	122	93 (76.2%)	12.16 (3.00)
Hyper/impulsive	12	10 (83.0%)	12.49 (4.62)
Whole ADHD sample	383	296 (77.3%)	11.56 (2.99)

*TD, typically developing. Although the two-sample *t*-tests comparing TD controls vs. ADHD (*p* = 0.001) and combined vs. inattentive (*p* = 0.003) indicate statistical differences in age, these differences were not relevant for classification (total scores of a logistic regression based solely on age are 0.50 and 0.49, respectively, i.e., very close to the scores by chance)*.

### Preprocessing of images

The preprocessing of the raw fMRI data was carried out by Cameron Craddock^2^ using scripts integrating the packages AFNI^3^, FSL^4^, and the Athena computational cluster at Virginia Tech’s ARC^5^. This pipeline was named “The Athena” and the preprocessed data is available at the Neurobureau website^6^. In order to assess quality metrics of the data, the mean and standard deviation of the volumes were calculated from all the scans and from all the subjects. These volumes were used to obtain maps of *z*-score. The absolute values of the *z*-scores were cut using a threshold value of three and summed across all intra-cranial voxels for a quality score for each image. This information is available at the link^7^. In this study, we considered the images from all labeled subjects provided by the ADHD-200 Competition because the quality metrics were satisfactory and also to allow comparisons of the performances with other studies based on the same dataset.

The main focus of The Athena pipeline is a systematic and homogeneous processing of all resting state fMRI datasets, and consisted on the following steps: exclusion of the first four echo-planar (EPI) volumes; slice timing correction; deoblique dataset; correction for head motion (first volume as reference); masking the volumes to exclude voxels at non-brain regions; averaging the EPI volumes to obtain a mean functional image; co-registration of this mean image to the respective anatomic image of the subject; spatial transformation of functional data into template space (4 mm × 4 mm × 4 mm resolution); extraction of BOLD (blood oxygenation level dependent) time series from white matter (WM) and cerebrospinal-fluid (CSF) using masks obtained from segmenting the structural data; removing effects of WM, CSF, motion, and trend using linear multiple regression and holding the residuals; temporal band-pass filter (0.009 < *f* < 0.08 Hz); spatial smoothing the filtered data using a Gaussian filter (full width at half maximum = 6 mm).

### Feature extraction from fMRI data

In classification problems, “features” are defined as the input/predictor variables which are used to generate class predictions (e.g., TD or ADHD) for new observations. Here, we evaluated three different types of features from functional brain imaging: ReHo, fALFF, and independent component analysis maps.

#### Regional homogeneity

The ReHo approach (Zang et al., 2004) is based on the calculation of Kendall coefficient of concordance between a voxel and its neighbor voxels. The basic idea of this method is to measure how similar are the BOLD signal of a given voxel and its neighbors, evaluating common local changes in different brain regions. In other words, ReHo analysis is a massive voxel-by-voxel analysis, for which, at each voxel, it is calculated the similarity between the BOLD from the central voxel and the voxels around it. This measure mirrors the spatial consistency of spontaneous activation (Zang et al., 2004; Biswal et al., 2010) at a brain region, and this is why it is named ReHo. In fact, the accurate neurophysiological interpretation of ReHo maps is still an open question. Some anatomical/functional features that may possibly influence ReHo coefficients are local atrophy and changes in local intrinsic connectivity. Several recent studies in literature have shown the potential value of ReHo in clinical applications (Qiu et al., 2011; Yan et al., 2011; Zhang et al., 2012), including ADHD (Zhu et al., 2008). Further details about technical issues (formulas, algorithms, and mathematical properties) can be found in the referred literature (Zang et al., 2004).

The ReHo maps from each subjects of ADHD-200 sample were provided by the Neurobureau and are available at the referred website^8^.

#### Fractional amplitude of low frequency fluctuations

Fractional amplitude of low frequency fluctuations approach was introduced by Zou et al. (2008) for the analysis of fMRI resting state datasets. Basically, the fALFF approach is a voxel-by-voxel calculation of low-frequencies spectral power (0.01–0.08 Hz) of BOLD signal. In other words, fALFF measures the relevance of low frequency fluctuations in the variance of the observed BOLD signal at each brain region. Although the neurophysiological processes which generate these fluctuations are not established, fALFF are usually interpreted as a measure of spontaneous activity during a resting state session (Zou et al., 2008). Similar to ReHo, several studies in the literature have explored the clinical value of fALFF (Hoptman et al., 2010; Han et al., 2011; Xuan et al., 2012). Further details about this method (formulas, algorithms, and mathematical properties) can be found in the referred literature (Zou et al., 2008).

The fALFF maps of ADHD-200 sample are available at the Neurobureau website.

#### Independent component analysis maps

Independent component analysis maps depicting functional connectivity were obtained using the approach proposed by Smith et al. (2009), in which the RSN were estimated using a modified dual regression approach. The RSN maps of each subject are available at the ADHD-200 website of Neurobureau. The analysis of the 10 first group ICA maps shows unequivocally that the fourth component is related to the default mode (positive values) and task-positive networks (negative values), which are frequently described as anti-correlated systems (Biswal et al., 2010). The fourth RSN map of each subject was then considered as a predictor feature for further classification, since the literature suggest association between these networks and ADHD (Castellanos et al., 2008; Fair et al., 2010).

#### Dimensionality reduction

The obtained number of predictor variables in fALFF, ReHo, and RSN maps were larger than the number of observations (subjects). Thus, it was necessary to apply a dimensionality reduction procedure in order to avoid numerical singularities (and in terms of computing time and optimization) and overfitting problems. Since neuroimaging data have a very high dimensionality, this step plays a crucial role in classification studies (Guyon and Elisseeff, 2003; De Martino et al., 2008). We applied the brain parcellation defined by the CC400 atlas, which was provided by the Neurobureau using the method developed by Craddock et al. (2012). This atlas was created using a functional parcellation of 400 regions of interest based on spectral clustering and spatial-clustering constraints, grouping neighbor voxels with similar frequencies power distribution from a dataset of 650 subjects. It contains parcellation of cortical and subcortical structures, and gray and WMs. The atlas is freely available at the link^9^. The main advantage of using this atlas, when compared to anatomical (AAL or Talairach) ones, is that the parcellation is based on functional properties and frequency-domain similarities instead of structural/morphological characteristics.

The mean value of the coefficients from fALFF, ReHo, and RSN fourth maps within each ROI defined by CC400 was calculated and then assumed to be the predictor variables in classification.

### Methods for classification

The main concern of the current study is the performance evaluation of different classifiers in predicting controls from ADHD subjects and inattentive vs. combined ADHD patients. Note that only the two-class prediction case was explored. We compared the following 10 classifiers (and abbreviator):

1. AdaBoostM1 (AdaB);
2. Bagging (Bagg);
3. LogitBoost (LogB);
4. L2-regularized L2-loss support vector classification dual (L2L2SVMd);
5. L2-regularized L2-loss support vector classification primal (L2L2SVMp);
6. L2-regularized L1-loss support vector classification dual (L2L1SVMd);
7. L1-regularized L2-loss support vector classification (L1L2SVMd);
8. L1-regularized logistic regression (L1logR);
9. L2-regularized logistic regression dual (L2logRd);
10. L2-regularized logistic regression (L2logR).

The first three classifiers are available in Weka package for data mining^10^ (Hall et al., 2009) and the others are implemented in liblinear library^11^ (Fan et al., 2008). The R platform for computational statistics^12^ was the environment chosen for implementing the classification procedures. The libraries RWeka and LiblineaR provided the integration between R (R Development Core Team, 2011) and the classification routines available in R public libraries. LiblineaR routines provide linear SVM and logistic regression with different penalization and optimization functions (and thus distinct sparsity in coefficients).

The AdaB (Freund and Schapire, 1996) is a machine learning approach based on boosting, which is a general method to improve the performance of classifiers. The basic idea is to repeatedly run a weak learning algorithm (e.g., classification trees) several times in an interactive manner. In an interactive algorithm, it increases the weights in observations which are hard to be learnt by the weak learning procedure. Bagg (Breiman, 1996) is an approach based on aggregating different versions from the same weak classifier (e.g., classification trees). Usually, multiple versions (multiple random samples) of the classifier are trained and the prediction is based on a plurality vote. The original study of Breiman (1996) have shown that Bagg can increase the accuracy and stability of the classifier. The LogB was introduced by Friedman et al. (1998) and it is a boosting algorithm with the same cost function of the logistic regression.

The other classifiers (from the LiblineaR package) are based on logistic regression, linear SVM, sparse logistic regression, and sparse SVM. The basic idea of linear SVM is to find a linear boundary with maximum separation margin between the two groups to be classified (see Alpaydin, 2010). The boundary is defined by a linear combination of the predictor variables and is founded in the structural risk minimization. This method was developed in order to increase the accuracy when predicting new observations (generalization power). Logistic regression fits a logistic function to the probability of group assignment. This logistic curve is a function of a linear combination of the predictor. Yamashita et al. (2008) and Ryali et al. (2010) have shown that logistic regression (mainly in its sparse version) can provide very accurate results in decoding distinct brain states.

Suppose for each subject *j* (from a total number of subjects *N*), we have a set of *k* predictor variables in a vector z _j_ =*x*_1,_ *x*_2,__…_ *x_k_* and the group of this subject is *y_j_* (specified by −1 or 1). The classifiers try to find the best set of coefficients in a vector w  which minimizes the quantity:

$$0.5*\text{w’w}+\sum_{i}^{N}L\left(\text{w};x_{i}y_{i}\right),$$

where *L* is called loss-function. Basically, the SVM, logistic regression and their variations (sparse solutions) differ on the specification of this function. The difference between primal and dual versions is not in the loss-function, but on the implementation of the optimization algorithm. A detailed explanation about each considered loss-functions and the optimization details can be found in Fan et al. (2008). In addition, the L1-regularized methods (loss-function) also include an embedded feature selection which penalizes features with low weights, setting them to zero, consequently, providing a sparse solution. Thus, the predictor features with non-null coefficients can be interpreted as the ones chosen by the classifier as containing discriminant information to separate the groups.

### Performance comparison

After image preprocessing and features extraction (Figure 1), the accuracy of each classifier was estimated. The evaluation of the extracted features and classifiers was carried out by using a total of 929 fMRI scans, i.e., 759 subjects released as the labeled data to train the classifiers, and the unlabelled scans of 170 subjects (group labels released after the competition) used to test the classifiers. In order to evaluate the stability and variability of the classifiers, the evaluation was carried out using Monte Carlo subsampling. At each iteration (from a total of 100), the data of 100 subjects from the 759 labeled subjects were randomly sampled without replacement. This subsample was then used as the training data for each of the 10 classifiers and to predict the label of the 170 subjects of the test data. This approach provided a set of measures of sensitivity and specificity at each iteration, which were then used to calculate descriptive statistics for each classifier. We chose Monte Carlo subsampling instead of leave-one-out cross-validation because the sample is relatively large, and thus, it may provide information about the stability (variability) of each classifier. Although the ADHD-200 sample is unbalanced, we prefer to train the classifiers using random sampling instead of constraining the groups to have the same size, because we believe this would be a more realistic scenario. The average sensitivity and specificity was calculated as an additional measure and we refer to them as the “total score.” The expected value of the total score in the case of classification by chance is 0.5. Regarding the combined vs. inattentive ADHD classification, the same pipeline and evaluation approach were used to classify the two subtypes. In this case, the sensitivity refers to the former group and specificity to the latter. In addition, the scores from the released test set (170 subjects) when training the classifiers using the whole training sample (759 subjects) were also computed.

### Identification of discriminative regions

To the best of our knowledge, there is no established approach to evaluate the relevance of each feature as containing predictive information. Furthermore, since each classifier is based on different algorithms and concepts, a general optimal approach applicable to all classifiers is not available. The current study is neither focused on analyzing how each classifier used each feature to generate the predictions nor on evaluating or comparing the relevance of each feature for each classifier. The main objective of this study was to evaluate the performance of different classifiers. However, the creation of brain maps depicting the relevant features, which are common to all classifiers, might provide insightful information about neural substrates of ADHD.

We tackle this issue by using a very simple and general approach, based on feature elimination and classifiers’ voting. All classifiers were trained using the full set of 759 subjects of the labeled data. The test data was then used to obtain predictions from each classifier and the final prediction was based on the most voted label. After computing the final prediction for each subject from the test data, the “global accuracy” (in the test data) was calculated. In the following, the classifiers were then re-trained using the training data but removing one feature at a time from the feature set. Then, the predictions for the test data were obtained and the “feature-out accuracy” based on classifiers vote was calculated. The difference between the feature-out and global accuracy was then considered to be the measure of the relevance of each feature left-out in discriminating the two groups of interest. Discriminative brain maps were then built to depict the 5% most relevant features from this general evaluation. When two or more modalities of features (e.g., ReHo and fALFF) are being considered as predictors in the same classifier, the results are displayed in separate brain maps for each modality for visualization purposes.

## Results

**Figure 2:**
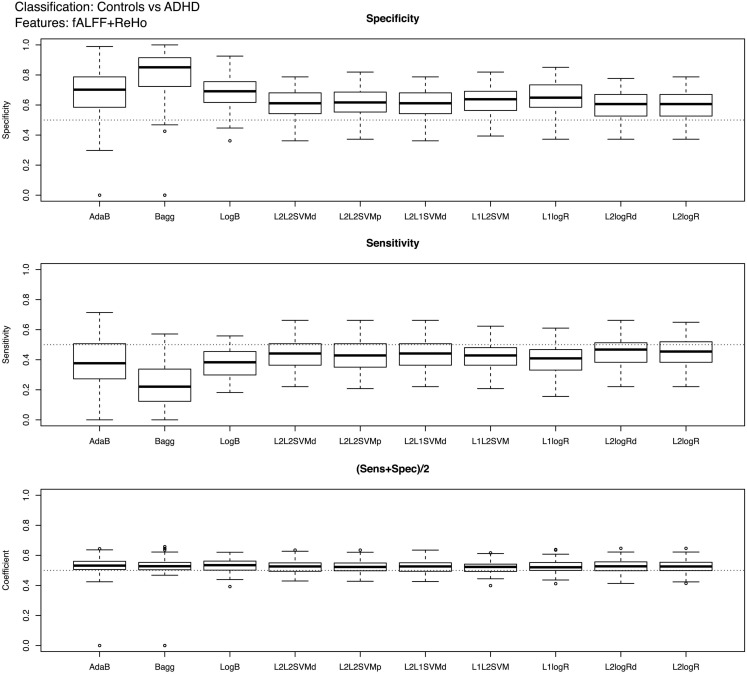
**Boxplots of the performance measures in control vs. ADHD patients classification when using fALFF+REHO features**.

**Figure 3:**
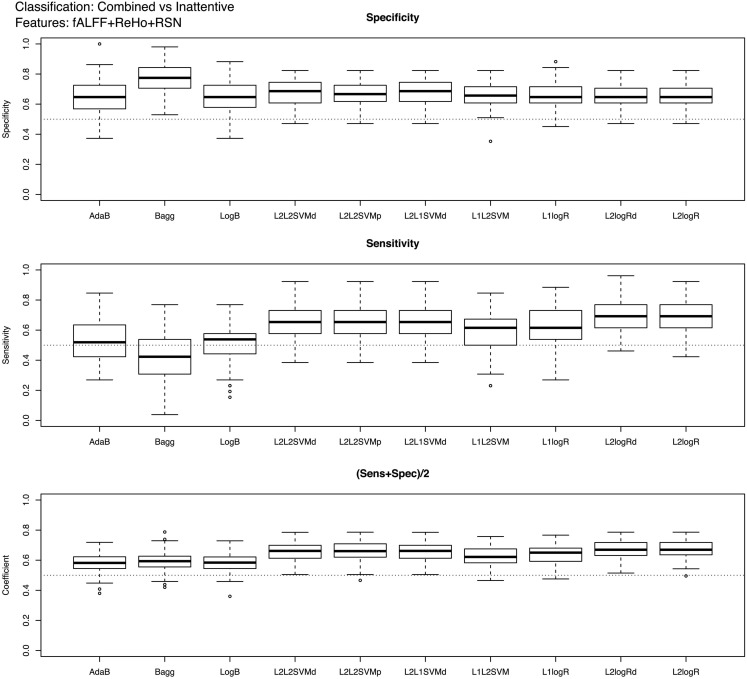
**Boxplots of the performance measures in combined vs. inattentive ADHD patients classification when using fALFF+REHO+RSN4 features**.

Boxplots showing the classifiers performance for controls vs. ADHD (features: ReHo+fALFF) and combined vs. inattentive ADHD (features: ReHo+fALFF+RSN4) predictions are presented in Figures 2 and 3, respectively (see Table 2; Tables A1 and A2 in Appendix for descriptive measures). The results of performance when using each feature modality separately can be found in Supplementary Material. Basically, Figures 2 and 3 show the results of the best combination of the features, when considering the total score.

**Table 2:** **Classification scores (sensitivity+specificity)/2 of the mean from Monte Carlo subsampling scores (and respective standard deviation) and when training the classifiers using all released training sample (759 subjects) and predicting the released test sample (170 subjects)**.

	Monte Carlo (%)	SD (%)	All training set (%)
**TD VS. ADHD**			
AdaB	53.6	3.9	55.5
Bagg	51.5	10.0	57.9
LogB	53.0	3.7	57.4
L2L2SVMd	52.7	3.6	52.3
L2L2SVMp	52.6	3.8	54.6
L2L1SVMd	52.7	3.6	52.3
L1L2SVM	52.7	3.5	55.8
L1logR	53.0	2.8	53.8
L2logRd	53.3	3.7	57.0
L2logR	53.2	3.8	57.0
**COMBINED VS. INATTENTIVE**			
AdaB	58.6	5.9	55.4
Bagg	58.5	7.1	59.3
LogB	58.4	7.2	60.4
L2L2SVMd	66.0	5.9	64.1
L2L2SVMp	66.0	5.9	63.2
L2L1SVMd	66.0	5.9	64.1
L1L2SVM	62.4	6.5	59.4
L1logR	64.3	6.0	63.2
L2logRd	67.0	5.6	65.1
L2logR	66.9	5.7	64.1

*These results are based on ReHo+fALFF features in TD vs. ADHD classification and ReHo+fALFF+RSN4 in combined vs. inattentive ADHD classification*.

In the case of controls vs. ADHD predictions, it was found that the ReHo and fALFF can separately (see Supplementary Material) and jointly (Figure 2) provide some information about the class of the subjects (median score near 0.54, but 75% of Monte Carlo resampling resulted in scores greater than 0.5, as shown by the boxplots). In addition, although the sensitivity and specificity measures were different between the classifiers (which is expected since one may compensate the other), the total scores were approximately the same independently of the method used. On the other hand, contrary to our expectation, the RSN4 was not relevant in this case, providing results near chance (median of total score near 0.5).

Regarding the results of combined vs. inattentive ADHD classification, the total score levels of all classifiers (Figure 3) were considerably higher than the controls vs. ADHD case (0.67 for L2logRd and L2logR). However, all three modalities (ReHo+fALFF+RSN4) were informative in this case, both separately as well as jointly (see Supplementary Material). In this case, the classifiers based on SVM and penalized logistic regression (from LiblineaR package) seem to have superior performance when compared to AdaB, Bagg, and LogB.

Complementary to the previous results, the scores of the classifiers trained with the entire training dataset of 759 subjects are presented in Table 2. Note that these scores are close to the mean scores of Monte Carlo subsampling described previously. In addition, Table 2 also suggests that the scores variability of Bagg in TD vs. ADHD classification was greater than the other methods. Although not in the scope of the current study, leave-one-subject-out and K-fold cross-validation results based on the whole sample (combining both released train and test sets in the same sample) are described in Table A3 in Appendix.

**Figure 4:**
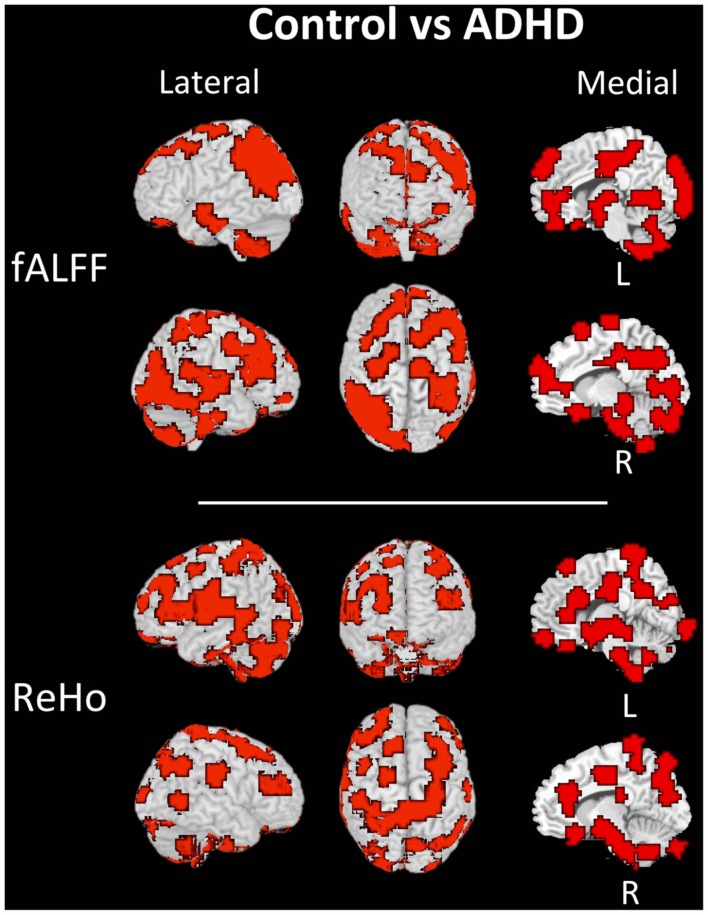
**Brain mapping of 5% regions containing most discriminative information in control vs. ADHD patients classification when using fALFF+REHO features**.

**Figure 5:**
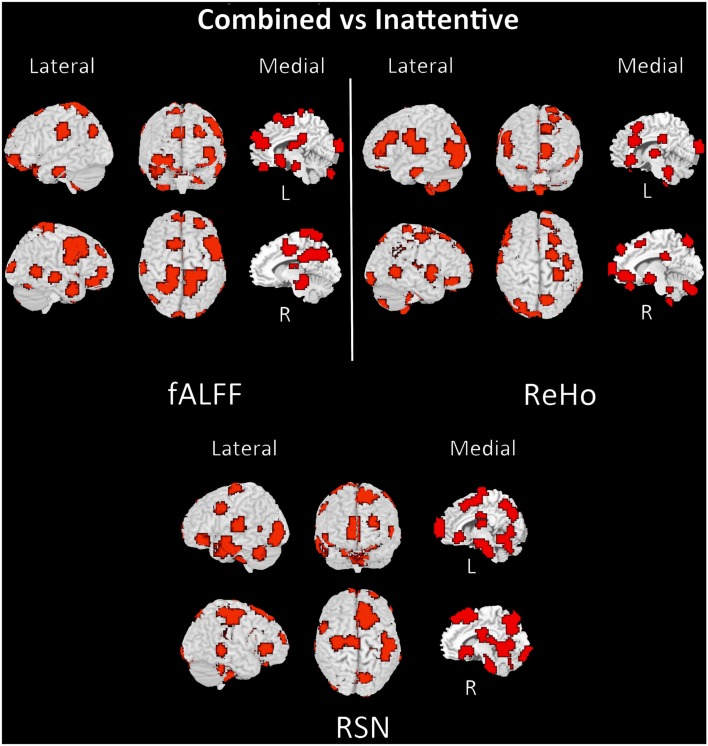
**Brain mapping of 5% regions containing most discriminative information performance in combined vs. inattentive ADHD patients classification when using fALFF+REHO+RSN4 features**.

The discriminant regions for controls vs. ADHD classification and combined vs. inattentive are shown in Figures 4 and 5, respectively. Apparently, in the control vs. ADHD case, the discriminative information seems to be distributed in several brain regions both for ReHo and fALFF maps. To compliment this, the RSN4, ReHo, and fALFF maps of combined vs. inattentive classification also depicts several spread brain regions, although the location of the discriminative information seems to be more focal and sparse than control vs. ADHD classification. However, each one of the three maps highlight different brain regions, suggesting that depending on the feature modality, the relevant information is not necessary in the same regions.

## Discussion

In the current study, we investigated the predictive accuracy of different machine learning and feature extraction algorithms for the classification of TD controls vs. ADHD patients and combined vs. inattentive ADHD. Our main concern was to compare the performance of the classifiers and also to evaluate the discriminant information contained in fALFF, ReHo, and RSN maps. We provided a systematic comparison of these methods using the ADHD-200 Consortium dataset released by the ADHD-200 competition (in September 2011).

The first point to be discussed is that the scientific community had a fundamental role in the implementation of the current study. The ADHD-200 consortium provided a large multicenter neuroimaging database and excluding some few exceptions, current studies exploring the predictive power of the state-of-the-art classifiers and neuroimaging in clinical applications report findings from relatively small samples (tens of subjects). In addition, the community has benefited considerably from the work of the Neurobureau and several collaborators, which preprocessed the enormous amount of raw data. These contributions were crucial since they allowed the participation of researchers from outside of the neuroimaging community and were extremely helpful to the researchers from the community, facilitating the features extraction implementation. The Neurobureau and the collaborators who provided the scripts for image processing motivated an innovative and multi/interdisciplinary research during the ADHD-200 competition. As described previously, the RSN, ReHo, fALFF maps, and CC400 atlas, which had a pivotal role in this study, were obtained via Neurobureau. In addition, the classification routines were also obtained from the scientific community via libraries LiblineaR and RWeka from the R-project platform for Computational Statistics.

The main findings of the current study can be summarized under two aspects: classifiers performance and identification of discriminative regions. The boxplots presented in Figure 2 suggest that the feature extraction and classification procedures considered were not effective in providing relevant information to distinguish ADHD patients from TD controls. Interestingly, all 10 classifiers had equivalent performance in this low accuracy rate, which may suggest that the chosen features were not highly discriminative at all. One finding which went contrary to our expectation was the poor accuracy of the classifiers when using the coefficients of RSN4 as features. Actually, we had chosen this feature inspired by the results of correlation analysis from Castellanos et al. (2008) and Weissman et al. (2006), who demonstrated that the posterior cingulate (PCC), one of the hubs in default-mode-network, has a pivotal role in attention maintenance and ADHD.

The classification between combined vs. inattentive ADHD (Figure 3) seems to be more promising and informative, not only because the prediction accuracies were considerably higher than the previous case but also because the performances of the tested classifiers were not equivalent. The best classifiers in this case were the L2-regularized logistic regression in dual (L2logRd) and L2-regularized logistic regression (L2logR), which are basically the same classifier but with a different implementation of parameters optimization. The median scores were both 0.67, indicating that the three features explored (ReHo, fALFF, and RSN from ICA maps) indeed contain relevant information to distinguish the two groups. Note that each one of these features (see boxplots in Supplementary Material) is discriminative not only in a joint analysis using the three modalities simultaneously as input to the classifier, but also in separate independent analysis. This is an important finding which reinforces the existence of quantitative and objective measures from functional brain images unveiling some characteristics of functional differences between the two subtypes of ADHD. Interestingly, both regularized SVM and regularized logistic regression methods provided higher accuracies when compared to Boosting and Bagg methods. We believe that this result can be explained by the fact that the former have more emphasis in structural risk minimization and in finding sparse solutions. Thus, they are more suitable in cases of high dimensionality. In other words, the way they handle overfitting problems results in better properties when predicting new observations.

The discriminant maps in Figures 4 and 5 were built in order to identify the regions from containing relevant information for classification. These figures highlight the regions that, if removed from the features set, will lead to a decrease in prediction accuracy in most classifiers. However, the brain mapping results suggest that the predictive information is not restricted to very few and sparse regions, but it is distributed over the whole brain. One of the advantages of machine learning classifiers when compared to massive-univariate *t*-tests or GLM is the ability to identify and use spatially distributed subtle differences to make predictions, even in cases in which different modalities (e.g., ReHo and fALFF) are combined (Ecker et al., 2010). In this sense, these findings point toward the investigations of multiple systems spread across the whole brain and not only the so called “blobology” confined to single or few regions, which is very common in neuroimaging literature. This is not an unexpected result, but additional studies are still necessary in order to obtain sufficient elements that are essential to the interpretation and comprehension of the implication of these findings. Since the main aims of the current study is the evaluation of clinical potential of features extraction and classifiers in ADHD and not answering a specific question or testing a neurobiological model, we chose to avoid elaborating conjectures or possible explanations about the regions highlighted in discrimination maps.

One of the limitations of this study and ADHD-200 sample has already been pointed out by the results of Alberta Team during the ADHD-200 competition: the demographic data (age, gender, IQ, etc) of the samples are not fully matched and thus, may contain discriminative information for group’s prediction. On the other hand, we argue that it would be unrealistic (and not meaningful from a clinical perspective) to have a complete homogeneous, controlled, and perfect matched samples, since it is already know that some demographic and behavioral data are indeed associated with ADHD. Actually, we also believe this is not a crucial point, so long as classification methods are not used as the main diagnostic reference but are used to provide complementary evidence and support. A second limitation to be mentioned is that we did not consider any brain region masking based on previous information from the literature. We chose to apply a more exploratory (whole brain) approach to investigate discriminant information in the data. However, the accuracy of the classifier could be higher if only the features from “*a priori*” regions known to be relevant were defined. Since the association between ADHD and the feature extraction methods are still in the initial stages, we prefer not to consider spatial constraints in our analysis. Interestingly, the classification results described in Table A3 in Appendix based on leave-one-subject-out and K-fold cross-validation using the whole sample (combining both train and test subjects released by the competition) indicate: (i) an increase in TD vs. ADHD score and a better performance of Weka classifiers, and (ii) a decrease in combined vs. inattentive score, when compared to the scores presented in Table 1. The results suggest an heterogeneity between train and test sets released by the competition. Details about the selection of train and test subjects were not provided by the competition but it was previously informed that the data from other sites would be part of the test set. Although the investigation of cohort effects and heterogeneity between train and test sets is relevant, in the current study, we limited the exploration to the context of ADHD-200 competition. Further exploration based on the whole sample and information about test subjects selection are extense but necessary to disentagle these issues. Finally, artifacts related to head and micro movements inside the scanner have been gaining attention in resting state research. Some studies (Power et al., 2012; Satterthwaite et al., 2012; Van Dijk et al., 2012) suggest that conventional motion-correction preprocessing are not sufficient to reduce bias in the analysis. The three features used in the current study were extracted using conventional motion-correction preprocessing (provided by Neurobureau).

The ADHD-200 completion is only the beginning of a new era of data and expertise sharing. Since the consortium has already released the complete data, we expect a big wave of studies using sophisticated data mining methods over the next few years. These studies will offer a detailed investigation of yet unexplored characteristics of neuroimaging data, which will play a crucial role to define more sensitive/specific features and also to develop new feature extraction methods. In this study, only three feature extraction methods were evaluated (ReHo, fALFF, and ICA) but other approaches can also be applied to the same data. In addition, although most classifiers have shown approximately the same performance, the accuracy was more dependent on the features, highlighting how crucial is the choice of the predictor variables. Finally, we hope that the ADHD-200 competition has inaugurated a new paradigm in Neuroscience research, strongly founded on public data sharing and interdisciplinary collaborations.

## Conflict of Interest Statement

The authors declare that the research was conducted in the absence of any commercial or financial relationships that could be construed as a potential conflict of interest.

## Supplementary Material

The Supplementary Material for this article can be found online at http://www.frontiersin.org/Systems_Neuroscience/10.3389/fnsys.2012.00068/abstract

## Footnotes

^1^http://fcon_1000.projects.nitrc.org/indi/adhd200/

^2^http://research.vtc.vt.edu/employees/cameron-craddock/

^3^afni.nimh.nih.gov/afni

^4^www.fmrib.ox.ac.uk/fsl/

^5^www.arc.vt.edu

^6^http://neurobureau.projects.nitrc.org/ADHD200

^7^http://www.nitrc.org/plugins/mwiki/index.php/neurobureau:AthenaPipeline

^8^http://www.nitrc.org/plugins/mwiki/index.php/neurobureau:AthenaPipeline

^9^http://www.nitrc.org/frs/downloadlink.php/3424

^10^www.cs.waikato.ac.nz/ml/weka

^11^www.csie.ntu.edu.tw/~cjlin/liblinear/

^12^www.r-project.org

## Appendix

**Table A1:** **Descriptive statistics of the performance measures in TD control vs. ADHD patients classification when using fALFF+REHO features**.

	Mean	SD	Median	Ql	Q3	Min	Max
**SPECIFICITY**							
AdaB	0.680	0.128	0.702	0.585	0.777	0.330	0.947
Bagg	0.807	0.181	0.851	0.766	0.907	0.000	1.000
LogB	0.679	0.102	0.681	0.625	0.747	0.404	0.915
L2L2SVMd	0.616	0.087	0.617	0.561	0.681	0.394	0.819
L2L2SVMp	0.626	0.086	0.617	0.572	0.691	0.404	0.830
L2L1SVMd	0.616	0.087	0.617	0.561	0.684	0.394	0.819
L1L2SVM	0.626	0.096	0.638	0.561	0.691	0.372	0.840
L1logR	0.649	0.102	0.665	0.585	0.713	0.383	0.840
L2logRd	0.602	0.089	0.617	0.543	0.662	0.394	0.809
L2logR	0.603	0.088	0.612	0.543	0.670	0.394	0.819
**SENSITIVITY**							
AdaB	0.392	0.127	0.396	0.286	0.484	0.143	0.688
Bagg	0.222	0.134	0.221	0.117	0.312	0.000	0.532
LogB	0.381	0.106	0.377	0.312	0.445	0.143	0.675
L2L2SVMd	0.438	0.072	0.429	0.390	0.494	0.182	0.610
L2L2SVMp	0.426	0.076	0.429	0.373	0.481	0.182	0.571
L2L1SVMd	0.438	0.072	0.429	0.390	0.494	0.182	0.597
L1L2SVM	0.428	0.087	0.429	0.364	0.497	0.234	0.636
L1logR	0.410	0.094	0.409	0.334	0.468	0.247	0.688
L2logRd	0.464	0.075	0.468	0.425	0.519	0.221	0.649
L2logR	0.462	0.073	0.455	0.416	0.506	0.221	0.649
**SCORE**							
AdaB	0.536	0.039	0.534	0.514	0.561	0.421	0.646
Bagg	0.515	0.100	0.533	0.502	0.560	0.000	0.631
LogB	0.530	0.037	0.532	0.510	0.550	0.435	0.628
L2L2SVMd	0.527	0.036	0.529	0.507	0.550	0.437	0.637
L2L2SVMp	0.526	0.038	0.526	0.503	0.549	0.435	0.633
L2L1SVMd	0.527	0.036	0.528	0.506	0.550	0.437	0.637
L1L2SVM	0.527	0.035	0.526	0.504	0.557	0.440	0.594
L1logR	0.530	0.028	0.531	0.507	0.549	0.462	0.630
L2logRd	0.533	0.037	0.534	0.508	0.561	0.442	0.605
L2logR	0.532	0.038	0.534	0.505	0.562	0.427	0.608

**Table A2:** **Descriptive statistics of the performance measures in combined vs. inattentive ADHD patients classification when using fALFF+REHO+RSN4 features**.

	Mean	SD	Median	Ql	Q3	Min	Max
**SPECIFICITY**							
AdaB	0.643	0.114	0.647	0.569	0.706	0.353	0.902
Bagg	0.760	0.117	0.765	0.667	0.843	0.373	0.961
LogB	0.645	0.094	0.647	0.588	0.706	0.373	0.863
L2L2SVMd	0.664	0.097	0.667	0.608	0.745	0.431	0.902
L2L2SVMp	0.660	0.090	0.667	0.588	0.725	0.451	0.902
L2L1SVMd	0.665	0.097	0.657	0.608	0.745	0.431	0.902
L1L2SVM	0.647	0.085	0.647	0.588	0.706	0.451	0.843
L1logR	0.665	0.093	0.667	0.608	0.745	0.451	0.902
L2logRd	0.647	0.094	0.647	0.588	0.706	0.412	0.902
L2logR	0.649	0.094	0.647	0.588	0.706	0.412	0.902
**SENSITIVITY**							
AdaB	0.528	0.166	0.519	0.423	0.625	0.115	0.923
Bagg	0.411	0.188	0.385	0.269	0.538	0.038	0.846
LogB	0.524	0.153	0.538	0.423	0.615	0.192	0.885
L2L2SVMd	0.656	0.123	0.654	0.577	0.769	0.308	0.885
L2L2SVMp	0.660	0.117	0.673	0.577	0.731	0.346	0.923
L2L1SVMd	0.655	0.122	0.654	0.577	0.769	0.308	0.885
L1L2SVM	0.601	0.138	0.615	0.538	0.692	0.192	0.923
L1logR	0.622	0.133	0.615	0.538	0.731	0.269	0.885
L2logRd	0.693	0.117	0.692	0.615	0.769	0.423	0.962
L2logR	0.689	0.117	0.692	0.615	0.769	0.423	0.962
**SCORE**							
AdaB	0.586	0.059	0.588	0.542	0.631	0.447	0.729
Bagg	0.585	0.071	0.585	0.535	0.642	0.429	0.727
LogB	0.584	0.072	0.589	0.544	0.631	0.399	0.737
L2L2SVMd	0.660	0.059	0.665	0.631	0.693	0.438	0.815
L2L2SVMp	0.660	0.059	0.669	0.629	0.689	0.438	0.844
L2L1SVMd	0.660	0.059	0.669	0.631	0.693	0.438	0.815
L1L2SVM	0.624	0.065	0.631	0.592	0.670	0.449	0.757
L1logR	0.643	0.060	0.651	0.602	0.689	0.468	0.748
L2logRd	0.670	0.056	0.670	0.641	0.708	0.515	0.834
L2logR	0.669	0.057	0.674	0.640	0.699	0.476	0.834

**Table A3:** **Classification scores [sensitivity + specificity]/2 of the leave-one-subject-out (LOSO) and k-fold cross-validation (30 subjects at each fold) when training the classifiers using all released subjects by ADHD-200 competition (train and test sets)**.

	LOSO (%)	k-fold (%)
**TD VS. ADHD**		
AdaB	63.4	59.3
Bagg	61.7	58.2
LogB	64.3	63.7
L2L2SVMd	59.0	60.4
L2L2SVMp	58.9	61.2
L2L1SVMd	59.6	60.1
L1L2SVM	59.9	61.2
L1logR	57.8	61.5
L2logRd	58.4	62.4
L2logR	58.4	62.4
**COMBINED VS. INATTENTIVE**		
AdaB	53.2	56.2
Bagg	60.2	58.4
LogB	58.7	53.0
L2L2SVMd	56.5	54.7
L2L2SVMp	58.0	55.0
L2L1SVMd	56.9	54.7
L1L2SVM	59.1	53.6
L1logR	61.3	54.7
L2logRd	57.6	55.5
L2logR	58.0	57.4

*These results are based on ReHo+fALFF features in TD vs. ADHD classification and ReHo+fALFF+RSN4 in combined vs. inattentive ADHD classification*.
